# Supplementary figures and images for: Transcriptome Analyses Revealed the Wax and Phenylpropanoid Biosynthesis Pathways Related to Disease Resistance in Rootstock-Grafted Cucumber
Source: Plants (Basel). 2023 Aug 16;12(16):2963. doi: 10.3390/plants12162963 (PMC10458401; doi:10.3390/plants12162963)

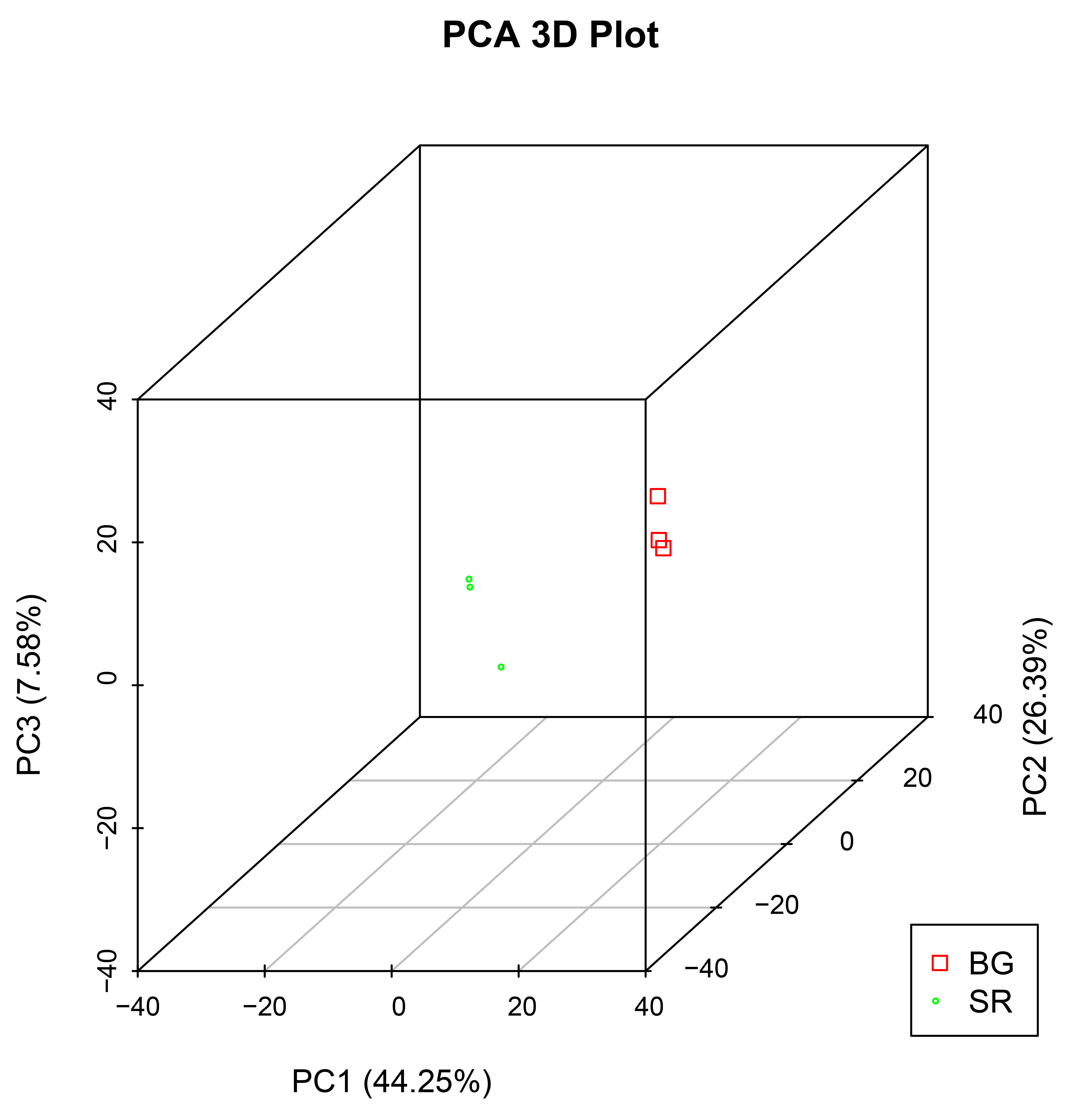

Supplement: Supplementary file 1 [file plants-12-02963-s001.zip › Figure S1.tif]
